# The chain-mediating role of workplace social capital and resilience in the relationship between change fatigue and proactive work behavior among nurses

**DOI:** 10.3389/fpsyg.2026.1972366

**Published:** 2026-10-08

**Authors:** Nan Yang, Qing Tian, Lin Nie

**Affiliations:** 1 General Practice and Emergency Medicine, Beijing Anzhen Nanchong Hospital of Capital Medical University & Nanchong Central Hospital, Nanchong, Sichuan, China; 2 Department of Radiology, Beijing Anzhen Nanchong Hospital of Capital Medical University & Nanchong Central Hospital, Nanchong, Sichuan, China

**Keywords:** chain mediation, change fatigue, nurses, proactive work behavior, resilience, workplace social capital

## Abstract

**Objective:**

To examine the associations among change fatigue, workplace social capital, resilience, and proactive work behavior in clinical nurses, and to test whether workplace social capital and resilience serially mediate the relationship between change fatigue and proactive work behavior.

**Methods:**

A cross-sectional survey was conducted from January to April 2026. Convenience sampling was used to recruit 1,018 clinical nurses. Participants completed a demographic questionnaire, the Change Fatigue Scale, the Resilience Scale for Nurses, and the Proactive Work Behavior Scale. Descriptive statistics, Pearson correlations, and a saturated path model with 5,000 bootstrap samples (AMOS 25.0) were used.

**Results:**

Nurses’ mean scores were 25.01 ± 7.97 for change fatigue, 77.50 ± 16.26 for workplace social capital, 63.80 ± 8.27 for resilience, and 31.78 ± 9.15 for proactive work behavior. Change fatigue was negatively correlated with workplace social capital, resilience, and proactive work behavior (*r* = −0.447 to −0.738, all *p* < 0.001). Workplace social capital and resilience were each associated with partial mediation (specific indirect effects −0.173, 24.9%; −0.187, 26.9%), and the serial indirect effect was also significant (−0.075, 10.8%). The direct effect accounted for 37.3% of the total effect. The four-factor measurement model fitted better than a one-factor model, but absolute fit was poor (RMSEA = 0.111) and discriminant validity involving resilience was not supported.

**Conclusion:**

Change fatigue is associated with lower proactive work behavior, and workplace social capital and resilience may partly explain this association. Monitoring change fatigue and fostering workplace social capital and resilience may support proactive work behavior; these propositions warrant longitudinal and intervention research.

## 1 Introduction

The 15th Five-Year Plan calls for deepening reforms in the healthcare sector and promoting the coordinated development of medical services (Liu et al., 2024). To better meet the public’s growing health needs, public hospitals must strengthen the advancement of medical technologies while also implementing comprehensive optimization and upgrades in process efficiency and service effectiveness. However, sustained reform requires healthcare professionals to continuously adapt and update their skills, which in turn places significant pressure and challenges on them. As a vital component of the healthcare industry, nurses are particularly susceptible to feelings of exhaustion and burnout—known as “change fatigue” (Bernerth et al., 2011)—due to frequent policy adjustments, technological innovations, and process reengineering. If prolonged change fatigue is not effectively alleviated, it may diminish nurses’ work vitality and motivation, thereby leading to a decline in service quality (Wang et al., 2026; Zeng et al., 2026). Therefore, alleviating nurses’ change fatigue and enhancing their work motivation is of paramount importance.

Proactive work behavior, as a key variable in positive psychology, primarily consists of three dimensions: individual proactive behavior, team member proactive behavior, and organizational member proactive behavior (Guo et al., 2022). Research shows (Yang et al., 2023; Chen et al., 2022) that high levels of proactivity are associated with high work performance, thereby generating greater benefits for the organization. Strengthening nurses’ proactivity is essential for promoting the organization’s overall development.

Workplace social capital refers to the social support network formed by nurses in their workplaces through mutual trust and support among colleagues (Sheingold and Sheingold, 2013). As a form of external resource, sufficient workplace social capital may help nurses to value their work and their own worth, and may thereby alleviate the fatigue associated with organizational change and support work motivation (Xu et al., 2020; Peng et al., 2026). These propositions are derived from theory rather than demonstrated causally in the present cross-sectional data. At the same time, social support theory posits that adequate support resources can alleviate negative emotions caused by stress and reduce the fatigue resulting from change.

Resilience, as an internal resource, primarily refers to a nurse’s ability to actively adapt when facing adversity and to quickly recover from stressful situations (Mealer et al., 2017). Strong resilience may enable nurses to demonstrate positive work adaptability, allowing them to overcome internal stress, resolve psychological issues, and actively engage in their work (Guo et al., 2018). However, existing research has primarily focused on whether nurses are engaged in their work rather than on their specific work behaviors, and the relationship between workplace social capital and individual resources at the level of resilience remains relatively underexamined.

Two complementary theoretical frameworks underpin the proposed model. First, according to the Conservation of Resources (COR) theory (Hobfoll, 1989), individuals strive to obtain, retain, and protect resources, and resource loss or the threat of loss produces stress and strain. Frequent organizational change is theorized to consume nurses’ psychological resources, which may give rise to change fatigue and reduce the resources available for proactive engagement. Second, according to the emotional exhaustion pathway (Maslach et al., 2001), sustained change-related demands deplete nurses’ energy, which may erode both the supportive relationships that constitute workplace social capital and the personal coping resources captured by resilience, ultimately reducing proactive work behavior. These frameworks jointly suggest that workplace social capital (an external resource) and resilience (an internal resource) may act as sequential mediators linking change fatigue to proactive work behavior. Accordingly, we formulated the following hypotheses: *H1*: Change fatigue is negatively associated with proactive work behavior. *H2*: Workplace social capital mediates the relationship between change fatigue and proactive work behavior. *H3*: Resilience mediates the relationship between change fatigue and proactive work behavior. *H4*: Workplace social capital and resilience serially mediate the relationship between change fatigue and proactive work behavior (i.e., change fatigue → workplace social capital → resilience → proactive work behavior).

A closely related study by Li et al. (2026) examined the mediating role of change fatigue between person–organization fit and proactive work behavior among nurses in oral and maxillofacial surgery departments in Sichuan Province. Our study differs from and extends this prior work in three ways: (1) we focus on change fatigue as the antecedent rather than as a mediator; (2) we model workplace social capital and resilience as sequential mediators, thereby addressing a chain of external and internal resources that prior work has not examined; and (3) we provide effect decomposition with bootstrap confidence intervals. These differences define the incremental contribution of the present study.

Based on this, the primary objective of this study is to test the associations between change fatigue and proactive behavior, while simultaneously exploring the mediating roles of workplace social capital and resilience. This research aims to provide a reference for efforts to reduce change fatigue among nurses, support their work motivation, and contribute to the delivery of efficient healthcare services to patients and their families.

## 2 Methods

### 2.1 Study participants

From January 2026 to April 2026, 1,018 clinical nurses were selected using convenience sampling as participants for this survey. Inclusion criteria: nurses practicing at the selected hospitals; engaged in frontline clinical nursing work for 1 year or longer; fully informed of the study content and voluntarily participating in the survey. Exclusion criteria: nurses undergoing standardized training; nurses on leave at the time of the survey.

The sample size was calculated using the formula: *n* = (*μα*/2 × *σ*/*δ*)^2^, with *α* set at 0.05 and a margin of error of *δ* = 1.0. In the pilot study for this research, the standard deviation for proactive behavior was 7.26. Taking into account a 20% attrition rate, the calculated sample size was 243; ultimately, 1,018 participants were included in the analysis. Because bootstrap-based mediation analysis and path analysis benefit from a larger sample (Preacher and Hayes, 2008; Schoenemann et al., 2017), and to compensate for the anticipated exclusion of invalid responses, a larger sample (*n* = 1,018) was collected. The large sample is a strength of the study.

Characteristics of the study participants: 79 male nurses (7.8%) and 939 female nurses (92.2%); Age: 300 participants (29.5%) aged ≤25, 423 participants (41.6%) aged 25–30, 214 participants (21.0%) aged 30–40, and 81 participants (8.0%) aged >40; Educational Level: 306 (30.1%) held an associate’s degree or lower; 712 (69.9%) held a bachelor’s degree or higher; Hospital Level: 682 (66.9%) worked at Grade III Class A hospitals; 212 (20.8%) at Grade III Class B hospitals; 124 (12.3%) at Grade II or lower hospitals; Permanent Staff Status: Yes, 102 (10.0%); No, 916 (90.0%); Professional Title: Junior, 534 (52.5%); Intermediate, 398 (39.1%); Senior, 86 (8.4%); Monthly personal income: ≤4,000 yuan, 200 (19.6%); 4,000–8,000 yuan, 642 (63.1%); >8,000 yuan, 176 (17.3%); Whether a certified nurse specialist: Yes, 111 (10.9%); No, 907 (89.1%); Willingness to work in nursing: Yes, 835 (82.0%); No, 183 (18.0%).

Participants were recruited from 8 hospitals in Nanchong, Sichuan Province, China, covering different hospital levels (Grade III Class A, Grade III Class B, and Grade II or lower). Hospitals were selected by convenience sampling: the research team contacted the nursing departments of the invited hospitals, which distributed the survey link to their clinical nurses.

This study was approved by the Medical Ethics Committee of the Nanchong Hospital of Beijing Anzhen Hospital, affiliated with Capital Medical University (Approval No. 2024(144)). All participants provided informed consent. The study was conducted in accordance with the Declaration of Helsinki; electronic informed consent was obtained from every participant before survey completion, responses were collected anonymously, and IP addresses were used solely to prevent duplicate submissions and were not linked to any personal identifiers.

### 2.2 Research instruments

#### 2.2.1 General information questionnaire

Designed by the research team. It included items such as the nurses’ age, educational level, gender, permanent staff status, average monthly income, and whether they were specialized nurses.

#### 2.2.2 Change fatigue scale

Developed by Bernerth et al. (2011) and adapted into Chinese by Zhang et al. (2024). The scale consists of a single dimension with six items. It employs a 7-point Likert scale, with scores ranging from 1 (“Strongly Disagree”) to 7 (“Strongly Agree”). The total score ranges from 6 to 42; a higher score indicates more severe change fatigue. The Cronbach’s α in the current sample was 0.979 and McDonald’s ω was 0.979; the α reported in the original Chinese adaptation study was 0.918 (Zhang et al., 2024).

#### 2.2.3 Resilience scale for nurses

Developed by Park et al. (2019) and adapted into Chinese by Yang et al. (2025). The scale comprises four dimensions—Personality (6 items), Situational (3 items), Relational (4 items), and Philosophical (6 items)—totaling 19 items. The scale uses a 5-point Likert scale, with scores ranging from 1 (“Hardly ever”) to 5 (“Almost always”). The total score ranges from 19 to 95; a higher score indicates a higher level of resilience. The Cronbach’s α in the current sample was 0.806 and McDonald’s ω was 0.797; the α reported in the original Chinese adaptation study was 0.881 (Yang et al., 2025).

#### 2.2.4 Workplace social capital scale

Developed by Sheingold and Sheingold (2013) and adapted into Chinese by Wu et al. (2025). The scale comprises four dimensions—external trust, solidarity, and empowerment (9 items); internal solidarity and trust (5 items); participation and affiliation (5 items); and social cohesion (3 items), comprising a total of 22 items. The scale uses a 5-point Likert scale, with scores ranging from 1 (“Hardly ever”) to 5 (“Almost always”); the total score ranges from 22 to 110. The Cronbach’s *α* in the current sample was 0.978 and McDonald’s ω was 0.945; the *α* reported in the original Chinese adaptation study was 0.922 (Wu et al., 2025).

#### 2.2.5 Proactive work behavior scale

Developed and revised by Chinese scholar Guo et al. (2022). It primarily consists of individual proactive behavior (3 items), team member proactive behavior (3 items), and organizational member proactive behavior (3 items), totaling 9 items. The scale uses a 7-point Likert scale, ranging from “Strongly Disagree” to “Strongly Agree,” scored 1–7, respectively. The total score ranges from 9 to 63. The Cronbach’s α in the current sample was 0.961 and McDonald’s ω was 0.948; the *α* reported in the original validation study was 0.916 (Guo et al., 2022). For consistency with the Abstract and the remainder of the text, the instrument is hereafter uniformly referred to as the Proactive Work Behavior Scale.

### 2.3 Data collection

This study employed an online survey via Questionnaire Star. The project team integrated the scales to develop the survey questionnaire based on the research objectives. Prior to the survey, all investigators underwent standardized training. The project leader sent the survey link to the relevant officials at the selected hospitals, who then forwarded the link to the nurses’ WeChat group and informed the participants of the survey’s purpose, significance, and instructions for completion. Finally, the research participants completed the questionnaire according to the provided instructions. The time required to complete the questionnaire ranged from 12 to 24 min, and each IP address was allowed only one response. Two pre-specified exclusion rules were applied. First, a questionnaire was excluded if the completion time was below 6 min. This threshold was fixed *a priori* and is lower than the estimated completion time of approximately 8 min stated in the questionnaire instructions, so that records completed in less than 6 min were judged unlikely to reflect careful item-by-item responding; among the retained records, completion times ranged from 12 to 24 min. Second, a questionnaire was excluded for patterned responding if it met either of the following pre-specified criteria: (a) the respondent selected the same response option for all 56 substantive items (straight-lining); or (b) the respondent produced a repeated alternating two-option sequence (e.g., 1-2-1-2-1-2…) across at least 30 consecutive items. These rules were fixed before data screening and were applied uniformly to all records. A total of 1,094 questionnaires were collected; 76 invalid questionnaires were excluded under the above rules, leaving 1,018 for final analysis, resulting in a valid response rate of 93.1%.

### 2.4 Statistical analysis

SPSS 23.0 software was used to analyze the exported data. Univariate normality was assessed using skewness and kurtosis; all four scale total scores satisfied the acceptable criteria (|skewness| < 2 and |kurtosis| < 7), supporting the use of Pearson correlation and maximum-likelihood-based path analysis. Continuous variables were described using mean and standard deviation, while categorical variables were described using frequencies and percentages. Cronbach’s α and McDonald’s ω were computed for each scale in the current sample; reliability coefficients from the original validation studies are also reported for comparison. Correlations were assessed using Pearson’s correlation analysis. Mediating effects were analyzed using AMOS 25.0. Specifically, a saturated path model with observed total scores (change fatigue → workplace social capital → resilience → proactive work behavior) was estimated by maximum likelihood; because the model is saturated (df = 0), global fit indices are not applicable, and we therefore report standardized and unstandardized path coefficients, standard errors, *R*^2^ values, and 5,000-sample percentile bootstrap 95% confidence intervals for the total, direct, total indirect, and specific indirect effects (Preacher and Hayes, 2008). We also examined the measurement structure with a four-factor confirmatory factor analysis (CFA) using subscale scores as indicators; model fit and discriminant validity were evaluated and are reported in Section 3.3, together with their implications for interpreting the resilience-related results. A *p*-value < 0.05 was considered statistically significant.

## 3 Results

### 3.1 Descriptive statistics

The survey results showed that nurses’ change fatigue scores were (25.01 ± 7.97) points, workplace social capital scores were (77.50 ± 16.26) points, resilience scores were (63.80 ± 8.27) points, and proactive behavior scores were (31.78 ± 9.15) points (see Table 1).

**Table 1:** Scores and reliability of the study variables (*n* = 1,018).

Variable	Items	Score range	Mean ± SD	Cronbach’s *α*	McDonald’s *ω*
Change fatigue	6	6–42	25.01 ± 7.97	0.979	0.979
Workplace social capital	22	22–110	77.50 ± 16.26	0.978	0.945
External trust, solidarity, and empowerment	9	9–45	31.40 ± 6.77		
Internal solidarity and trust	5	5–25	18.19 ± 4.15		
Participation and affiliation	5	5–25	17.96 ± 4.19		
Social cohesion	3	3–15	9.95 ± 2.38		
Resilience	19	19–95	63.80 ± 8.27	0.806	0.797
Personality profile	6	6–30	18.52 ± 3.56		
Situational model	3	3–15	10.93 ± 1.44		
Relational model	4	4–20	13.26 ± 2.21		
Philosophical model	6	6–30	21.08 ± 3.42		
Proactive work behavior	9	9–63	31.78 ± 9.15	0.961	0.948
Individual proactive behavior	3	3–21	10.13 ± 3.36		
Team member proactive behavior	3	3–21	10.94 ± 3.14		
Organizational member proactive behavior	3	3–21	10.72 ± 3.13		

*α* and *ω* values are reliability coefficients computed in the current sample. Bold values indicate the internal-consistency reliability coefficients (Cronbach’s α and McDonald’s ω) for the total scale and its dimensions.

### 3.2 Correlation analysis

The results of this study show that the total score for nurses’ change fatigue was negatively correlated with workplace social capital, resilience, and proactive behavior—both in terms of total scores and scores on each dimension (*r* = −0.738 to −0.387, all *p* < 0.001); the total scores and dimension scores for workplace social capital were positively correlated with those for resilience and proactive behavior (*r* = 0.406–0.709, all *p* < 0.001); the total scores and subscale scores for resilience were positively correlated with those for proactive behavior (*r* = 0.667–0.716; all *p* < 0.001).

Table 2 reports the Pearson correlation matrix for the four total scores with 95% confidence intervals; all correlations were statistically significant (*p* < 0.001).

**Table 2:** Pearson correlations among the study variables (*n* = 1,018).

Variable	1	2	3	4
1. Change fatigue	—			
2. Workplace social capital	−0.447 [−0.502, −0.392]	—		
3. Resilience	−0.738 [−0.780, −0.697]	0.709 [0.666, 0.753]	—	
4. Proactive work behavior	−0.606 [−0.655, −0.557]	0.659 [0.612, 0.705]	0.716 [0.673, 0.759]	—

Values are Pearson r with 95% confidence interval in brackets; all *p* < 0.001.

### 3.3 Common method bias and measurement validity

To examine whether the data exhibited severe common method bias, we first conducted Harman’s single-factor test on all 56 items. The results showed 11 factors with eigenvalues greater than 1, and the variance explained by the first factor was 44.534%. Because the first-factor variance exceeded the conventional 40% threshold, we did not rely on Harman’s test alone to rule out common method variance. Instead, we implemented several procedural controls (anonymous administration, counterbalanced item ordering, and clear instructions emphasizing that there were no right or wrong answers) and conducted a CFA comparison of the proposed four-factor model with a one-factor model using identical indicators. The four-factor model fit the data significantly better than the one-factor model (Δ*χ*^2^ = 6,903.1, Δdf = 6, *p* < 0.001; four-factor model: CFI = 0.936, TLI = 0.923, RMSEA = 0.111; one-factor model: CFI = 0.621, RMSEA = 0.262), indicating that the four-factor structure was relatively superior to the one-factor structure. This relative superiority does not establish adequate absolute fit, and—as reported below and in Supplementary Table S1—discriminant validity involving resilience was not supported. Nonetheless, because all variables were self-reported at a single time point, common method variance cannot be entirely excluded and is therefore acknowledged as a limitation.

In addition, confirmatory factor analysis (CFA) of the four-factor measurement model (six change-fatigue items; four, four, and three subscale scores for workplace social capital, resilience, and proactive work behavior, respectively) yielded *χ*^2^ = 1,523.5, df = 113, CFI = 0.936, TLI = 0.923, GFI = 0.931, AGFI = 0.917, NFI = 0.931, RMSEA = 0.111 [90% CI (0.105, 0.117)], and SRMR = 0.055. The RMSEA of 0.111 indicates substantial absolute misfit and exceeds the conventional 0.08 cutoff; its 90% confidence interval [0.105, 0.117] does not include 0.08. The incremental indices (CFI = 0.936, TLI = 0.923, GFI = 0.931, AGFI = 0.917, NFI = 0.931) and SRMR = 0.055 were acceptable, but a model with acceptable incremental fit and unacceptable absolute fit should not be interpreted as having adequate overall measurement fit. The four-factor model was relatively superior to the one-factor model (reported in the preceding paragraph), yet absolute misfit remained substantial and discriminant validity involving resilience was not supported (see the next paragraph and Supplementary Table S1). Accordingly, the measurement findings should be interpreted as indicating that a four-factor structure fits better than a one-factor structure, but not as establishing strong measurement quality.

Standardized factor loadings, average variance extracted (AVE), and composite reliability (CR) for the four-factor model are reported in Supplementary Table S1. Loadings were strong for change fatigue (0.896–0.973), workplace social capital (0.841–0.990), and proactive work behavior (0.876–0.962), and moderate for resilience (0.604–0.764). AVE met the ≥0.50 criterion for change fatigue (0.882), workplace social capital (0.805), and proactive work behavior (0.857), but was below the criterion for resilience (0.494); CR exceeded 0.70 for all constructs (0.795–0.978), indicating adequate-to-strong reliability. Standardized factor correlations were: change fatigue with workplace social capital *r* = −0.469, with resilience *r* = −0.821, and with proactive work behavior *r* = −0.607; workplace social capital with resilience *r* = 0.819, with proactive work behavior *r* = 0.662; and resilience with proactive work behavior *r* = 0.834. Discriminant validity assessed by the heterotrait–monotrait ratio (HTMT) was acceptable for the three pairs not involving resilience (change fatigue–workplace social capital 0.556, change fatigue–proactive work behavior 0.698, workplace social capital–proactive work behavior 0.797), but exceeded the 0.90 criterion for all three pairs involving resilience (change fatigue–resilience 1.24, workplace social capital–resilience 1.30, resilience–proactive work behavior 1.28). Discriminant validity involving resilience was therefore not supported, and resilience overlapped substantially with the other three constructs at the latent level. Consequently, the resilience-specific estimates should be interpreted cautiously. This pattern is consistent with the multidimensional nature of the Resilience Scale for Nurses and with its borderline AVE (0.494).

### 3.4 Chain mediation analysis

A chain mediation model was constructed with change fatigue as the independent variable, workplace social capital as mediator M1, resilience as mediator M2, and proactive behavior as the dependent variable. Because the model was specified with observed total scores, the path model is saturated (df = 0) and global fit indices are not applicable; we therefore report path coefficients, *R*^2^ values, and bootstrap confidence intervals instead. After 5,000 bootstrap samples, none of the 95% confidence intervals for the total, direct, total indirect, or specific indirect effects included zero, indicating that all effects were statistically significant.

The results show that the direct negative effect of change fatigue on proactive behavior is −0.260 (standardized *β* = −0.226), accounting for 37.3% of the total effect; the mediating effect of workplace social capital between change fatigue and proactive behavior was −0.173 (standardized *β* = −0.151), accounting for 24.9% of the total effect; the mediating effect of resilience was −0.187 (standardized *β* = −0.163), accounting for 26.9% of the total effect; the chained mediating effect of workplace social capital and resilience was −0.075 (standardized *β* = −0.066), accounting for 10.8% of the total effect (see Table 3). The *R*^2^ values were 0.200 for workplace social capital, 0.725 for resilience, and 0.581 for proactive work behavior (see Figure 1).

**Table 3:** Chain-mediation effects of workplace social capital and resilience on the relationship between change fatigue and proactive work behavior (*n* = 1,018).

Effect type	Unstd. estimate	Std. *β*	Proportion (%)	95% CI (bootstrap)
Total effect	−0.695	−0.606	100.0	[−0.752, −0.640]
Direct effect	−0.260	−0.226	37.3	[−0.335, −0.187]
Total indirect effect	−0.436	−0.380	62.7	[−0.499, −0.374]
CF → WSC → PB	−0.173	−0.151	24.9	[−0.217, −0.135]
CF → RES → PB	−0.187	−0.163	26.9	[−0.234, −0.139]
CF → WSC → RES → PB	−0.075	−0.066	10.8	[−0.099, −0.053]

CF, Change Fatigue; WSC, Workplace Social Capital; RES, Resilience; PB, Proactive Work Behavior. Confidence intervals are percentile bootstrap intervals based on 5,000 resamples. Path coefficients are unstandardized (B): a1 (CF → WSC) = −0.912, a2 (CF → RES) = −0.547, d21 (WSC → RES) = 0.241, b1 (WSC → PB) = 0.190, b2 (RES → PB) = 0.342, and c′ (CF → PB) = −0.260 (all *p* < 0.001). Standardized coefficients for the direct and indirect effects are reported in the text.

**Figure 1:**
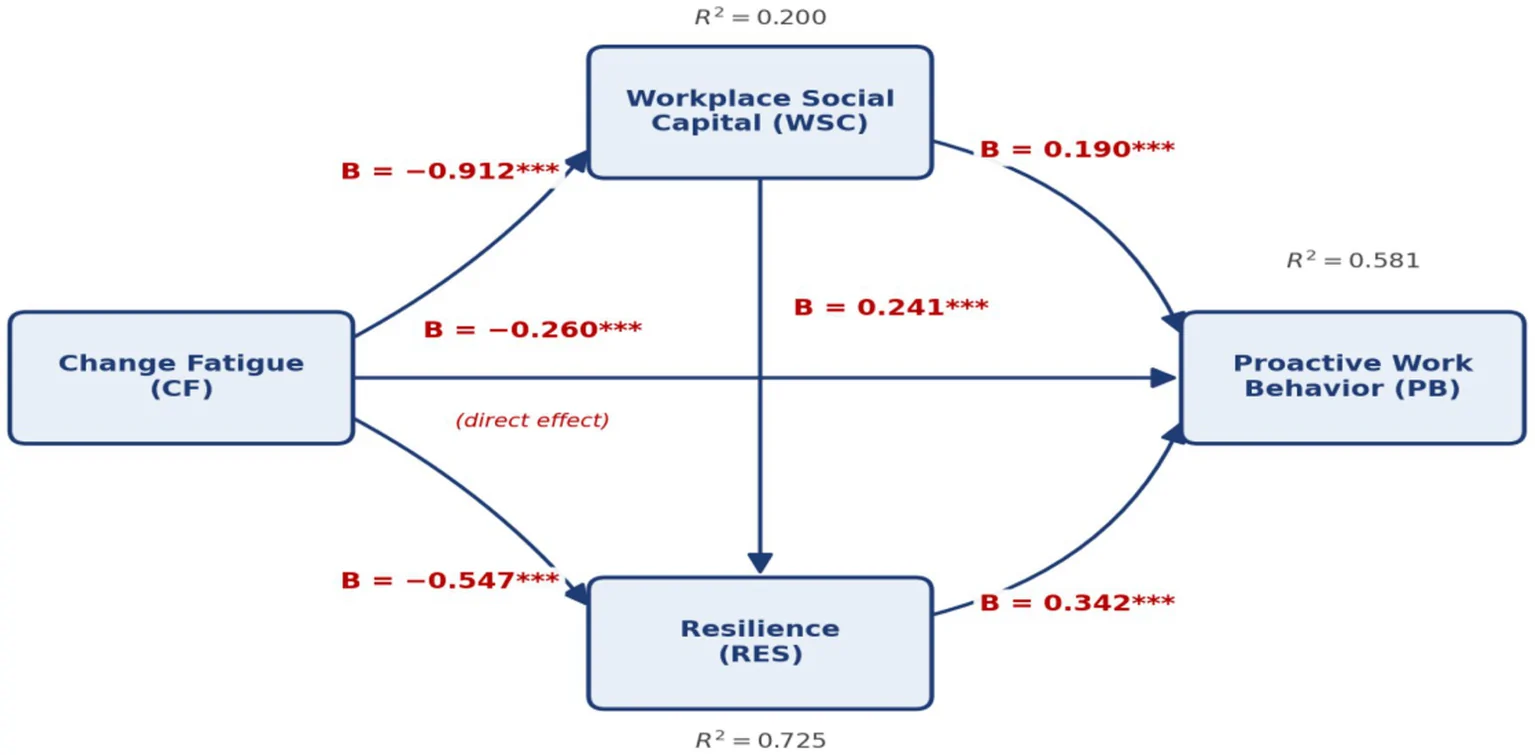
Chain-mediation model of the relationships among change fatigue, workplace social capital, resilience, and proactive work behavior. Unstandardized path coefficients (*B*) are shown; ***p < 0.001.

## 4 Discussion

### 4.1 Descriptive findings

The results of this study show that nurses’ proactive behavior scores averaged (31.78 ± 9.15) points, indicating a moderate-to-slightly-below-midpoint level (the total-score midpoint for the 7-point scale is 36). Because no validated population norm or clinically meaningful threshold exists for the Proactive Work Behavior Scale, this interpretation is descriptive and relative to the scale midpoint and should not be read as a normative judgment. In this study, 66.9% of the nurses worked in regional general hospitals; it is possible that patients in such hospitals tend to have more complex and severe conditions, which could be associated with a relatively heavy workload, and that nurses in these hospitals may experience intense competition that contributes to a heavier workload and may be associated with greater susceptibility to work-related fatigue and, consequently, lower work motivation (Zhang and He, 2023). These possibilities are offered as hypotheses for future research rather than as demonstrated mechanisms. Furthermore, as patients’ healthcare needs continue to rise, nurses must not only possess solid professional skills but also demonstrate a strong service orientation and corresponding capabilities.

The results of this study show that nurses’ workplace social capital score was (77.50 ± 16.26) points, which is at the upper-middle level compared to the scale’s midpoint. One possible explanation is that an increasing number of hospitals and departments may emphasize a “magnetic” management style, in which frontline nurses may receive care and support from both their departments and the organization as a whole, potentially forming a supportive network and being associated with higher workplace social capital (Zhang et al., 2025). This attribution is speculative: management style was not measured in this study, and the explanation cannot be tested with the present data. These interpretations of “upper-middle” or “slightly above average” are descriptive comparisons with the scale midpoint and should be interpreted cautiously in the absence of normative reference data.

The results of this study show that nurses’ resilience scores averaged (63.80 ± 8.27) points, placing them at a slightly above-average level compared to the scale’s midpoint. Furthermore, 69.9% of the nurses surveyed in this study held a bachelor’s degree or higher. It is possible that nurses with higher educational attainment generally possess stronger learning abilities and are able to continuously improve their stress-adaptation skills through appropriate learning channels, which could contribute to relatively higher resilience scores; this explanation is tentative because educational attainment was not examined as a predictor in the present analysis.

The results of this study show that nurses’ change fatigue scores were (25.01 ± 7.97) points, which, compared to the midpoint of the scale, is at a slightly above-average level and higher than the findings reported in Hai et al. (2026). We note that Hai et al. (2026) used a different version or administration of the Change Fatigue Scale, so the comparison should be interpreted cautiously. As the healthcare industry continues to develop, hospitals will implement corresponding policies and improve relevant processes. For nurses on the front lines of clinical care, frequent policy changes, process restructuring, and optimization may contribute to change-related fatigue and exhaustion (Havaei et al., 2021). Because these explanatory factors (e.g., staffing shortages, workload, competition, and psychological support programs) were not measured in this study, they should be regarded as tentative hypotheses for future research rather than demonstrated explanations.

### 4.2 Change fatigue and proactive work behavior

The results of this study show that change fatigue was negatively correlated with proactive behavior; nurses with higher levels of change fatigue reported lower levels of proactive behavior. Consistent with the cross-sectional design, this association does not imply a causal ordering. On the one hand, frequent organizational changes may require nurses to devote more time and effort to continuously adapt to process revisions and policy implementation, potentially increasing their workload and making it difficult for them to maintain a positive mindset at work. On the other hand, according to the theory of emotional exhaustion, when nurses experience fatigue due to frequent organizational changes, the psychological burden may lead to emotional exhaustion, which could in turn be associated with behavioral withdrawal and lower proactive behavior (Zhang et al., 2026). These mechanisms should be tested in future longitudinal or experimental studies.

### 4.3 The mediating roles of workplace social capital and resilience

Workplace social capital was associated with a partial mediating role between nurses’ change fatigue and proactive behavior. This indicates that part of the association between change fatigue and proactive behavior may operate through workplace social capital; it does not establish that enhancing workplace social capital will causally reduce change fatigue. Workplace social capital primarily refers to the social support networks and resources available to nurses within their organizations and workplaces. When nurses receive care and support from their organization, leaders, and colleagues, their intrinsic adaptive capacity and stress regulation abilities may increase. This may equip them with the capabilities and positive mindset to manage the fatigue resulting from frequent organizational changes and to adapt to organizational transformations, thereby potentially manifesting as vitality in their work and relatively high scores on proactive behavior (Zhang, 2023). These interpretations are offered as plausible explanations consistent with social support theory and should be verified in intervention studies.

Resilience was associated with a partial mediating role between nurses’ change fatigue and proactive behavior. That is, resilience may account for part of the association between change fatigue and proactive behavior, although causal ordering cannot be established from cross-sectional data. This may be because nurses with high resilience possess emotional regulation and stress-coping abilities, which may buffer the work overload and stress resulting from organizational change, thereby reducing feelings of fatigue and helping to maintain enthusiasm and vitality at work. Conversely, nurses with low resilience may be more prone to perceiving organizational changes—such as process reengineering and policy revisions—as sources of stress and burden, which may further exacerbate the negative emotions arising from change and be associated with lower levels of proactive behavior (Fan et al., 2023). Given that discriminant validity involving resilience was not supported, these resilience-specific interpretations should be regarded as tentative.

Workplace social capital and resilience were associated with a chained mediating role between nurses’ change fatigue and proactive behavior. This suggests that the association between change fatigue and proactive work behavior may operate through a sequential pathway in which change fatigue is associated with lower workplace social capital, which in turn is associated with lower resilience and thus with less proactive behavior. According to the mechanism of action outlined in Conservation of Resources theory, frequent organizational change may contribute to change fatigue among nurses, potentially causing emotional and physical exhaustion that leaves them with little time or energy to participate in organizational activities. This may reduce communication and collaboration with colleagues and supervisors, which in turn may lower perceived support and care and contribute to a decline in workplace social capital (Liu et al., 2025). When low workplace social capital manifests as a state of deprivation, nurses—lacking individuals to confide in and sources of support—may have no appropriate channels for support when facing the fatigue caused by organizational change. Consequently, their ability to cope with stress on their own may diminish, potentially reducing their resilience levels. Furthermore, a decline in resilience may hinder nurses’ ability to recover quickly from the challenges of change fatigue, potentially making them prone to negative cognitions and energy depletion; consequently, their positive work attitudes may diminish, their concentration may wane, and their proactive behavior may decrease. Because latent discriminant validity involving resilience was not supported, this serial pathway should be regarded as a tentative theoretical model rather than an established mechanism.

The present findings are consistent with the hypothesized serial-mediation structure at the level of cross-sectional associations, but they cannot validate a causal or temporal pathway. Before implementing process improvements or organizational changes, managers could consider clearly articulating the benefits and necessity of the change to ensure that frontline nurses have a correct understanding of and are psychologically prepared for it; at the same time, managers could consider establishing a tiered support system. Through mutual aid groups among departments and colleagues, as well as change-sharing sessions and stress-relief forums, such initiatives may help to foster trust and support among colleagues, strengthen the development of psychosocial capital, and support nurses’ stress coping and adaptive abilities. These management recommendations should be regarded as propositions to be tested in longitudinal or intervention research, rather than as effects demonstrated by this cross-sectional survey.

## 5 Limitations

This study has several limitations. First, the cross-sectional design limits causal inference between variables—a limitation acknowledged in international methodological guidelines for mediation research (Maxwell and Cole, 2007; Preacher and Hayes, 2008). In particular, the proposed temporal ordering (change fatigue → workplace social capital → resilience → proactive work behavior) cannot be established from simultaneously collected data, and reverse or reciprocal models are equally plausible. Longitudinal or experimental designs are required to test the direction of these associations and to control for unmeasured confounding. Second, all data were self-reported, which may introduce social desirability bias and common method variance; although procedural controls were implemented and the four-factor model fitted better than the one-factor model, discriminant validity involving resilience was not supported and common method variance cannot be entirely excluded. Third, the measurement model showed substantial absolute misfit (RMSEA = 0.111), and HTMT values involving resilience (1.24–1.30) exceeded the criterion; the resilience-specific results should therefore be interpreted cautiously, and future work should examine the factor structure of the Resilience Scale for Nurses more closely. Fourth, convenience sampling may affect sample representativeness, and the generalizability of the findings to other hospitals and healthcare systems should be considered cautiously. Fifth, because respondents’ hospital and ward membership was not retained, hospital-level intraclass correlation coefficients and design effects could not be estimated, and any underestimation of standard errors resulting from within-hospital dependence could not be assessed; the independence assumption could not be formally tested. Future studies that retain hospital and ward identifiers should apply multilevel modeling or cluster-robust standard errors to address this issue.

## 6 Conclusion

This study provides cross-sectional evidence of the associations among nurses’ change fatigue, workplace social capital, resilience, and proactive work behavior, and is consistent with the serial-mediating roles of workplace social capital and resilience in these associations. The findings should be interpreted with the following qualifications: the four-factor measurement model fitted better than a one-factor model, but absolute fit was poor, and discriminant validity involving resilience was not supported; therefore, the resilience-related findings require confirmation in future studies. The observed-total-score indirect effects are compatible with the specified statistical model, but they do not validate separate latent mechanisms or a temporal/causal chain. These findings provide a reference for developing longitudinal studies and intervention programs aimed at the decline in proactive behavior associated with change fatigue; however, causal conclusions require future longitudinal or experimental research.

## Data Availability

The original contributions presented in the study are included in the article/Supplementary material, further inquiries can be directed to the corresponding author.

## References

[ref1] Bernerth J. B. Walker H. J. Harris S. G. (2011). Change fatigue: development and initial validation of a new measure. Work Stress. 25, 321–337. doi: 10.1080/02678373.2011.634280

[ref2] Chen W. Li N. Han L. Luo L. Yin F. (2022). Correlations among job competence, self-efficacy, and work performance among frontline clinical nurses. Chin. J. Health Stand. Manag. 13, 18–22.

[ref3] Fan Y. Xu S. Tan Y. Li X. (2023). Research progress on change fatigue in the nursing field. Chin. Nurs. Manag. 23, 1873–1877. doi: 10.3969/j.issn.1672-1756.2023.12.021

[ref4] Guo Y. F. Luo Y. H. Lam L. Cross W. Plummer V. Zhang J. P. (2018). Burnout and its association with resilience in nurses: a cross-sectional study. J. Clin. Nurs. 27, 441–449. doi: 10.1111/jocn.14052 28677270

[ref5] Guo T. Shang S. Chen Y. (2022). Test of measurement equivalence for the proactive behavior scale in Chinese organizations. Psychol. Res. 15, 432–438. doi: 10.19988/j.cnki.issn.2095-1159.2022.05.006

[ref6] Hai H. Li F. Chen G. Zhang Y. (2026). Analysis of the latent profile and influencing factors of change fatigue among clinical nurses. J. Nurs. 41, 62–66.

[ref7] Havaei F. Ma A. Staempfli S. MacPhee M. (2021). Nurses’ workplace conditions impacting their mental health during COVID-19: a cross-sectional survey study. Healthcare 9:84. doi: 10.3390/healthcare9010084, 33467080 PMC7830057

[ref8] Hobfoll S. E. (1989). Conservation of resources: a new attempt at conceptualizing stress. Am. Psychol. 44, 513–524. doi: 10.1037/0003-066X.44.3.513, 2648906

[ref9] Li X. Pu J. Yang X. Zhao J. (2026). The mediating role of change fatigue among nurses in oral and maxillofacial surgery in Sichuan Province between person-organization fit and proactive work behavior. Occup. Health 42, 1511–1516. doi: 10.13329/j.cnki.zyyjk.2026.0279

[ref10] Liu Z. Yan X. Wang Z. (2025). The effect of nurses’ organizational support on turnover intention: the chain-mediating role of psychological resilience and change fatigue. Front. Psychol. 16:1696053. doi: 10.3389/fpsyg.2025.1696053, 41439224 PMC12719498

[ref11] Liu X. Yao X. Qian Q. Hu M. Wang X. Zhao L. . (2024). Development and application of a performance evaluation data monitoring and management system for tertiary public hospitals. Mod. Hosp. 24, 434–437. doi: 10.3969/j.issn.1671-332X.2024.03.029

[ref12] Maslach C. Schaufeli W. B. Leiter M. P. (2001). Job burnout. Annu. Rev. Psychol. 52, 397–422. doi: 10.1146/annurev.psych.52.1.397, 11148311

[ref13] Maxwell S. E. Cole D. A. (2007). Bias in cross-sectional analyses of longitudinal mediation. Psychol. Methods 12, 23–44. doi: 10.1037/1082-989X.12.1.23, 17402810

[ref14] Mealer M. Jones J. Meek P. (2017). Factors affecting resilience and the development of posttraumatic stress disorder in critical care nurses. Am. J. Crit. Care 26, 184–192. doi: 10.4037/ajcc2017798 PMC5685839 28461539

[ref15] Park S. Choi M. Kim S. (2019). Validation of the resilience scale for nurses (RSN). Arch. Psychiatr. Nurs. 33, 434–439. doi: 10.1016/j.apnu.2019.08.004 31280791

[ref16] Peng X. Liu S. Ou Y. Zhu W. Liang J. Yang L. . (2026). A study on the mediating effect of workplace social capital among oncology nurses on the relationship between person-organization fit and clinical leadership. Mod. Clin. Nurs. 25, 79–86.

[ref17] Preacher K. J. Hayes A. F. (2008). Asymptotic and resampling strategies for assessing and comparing indirect effects in multiple mediator models. Behav. Res. Methods 40, 879–891. doi: 10.3758/BRM.40.3.879, 18697684

[ref18] Schoenemann A. M. Boulton A. J. Short S. D. (2017). Determining power and sample size for simple and complex mediation models. Soc. Psychol. Personal. Sci. 8, 379–386. doi: 10.1177/1948550617715068

[ref19] Sheingold B. H. Sheingold S. H. (2013). Using a social capital framework to enhance measurement of the nursing work environment. J. Nurs. Manag. 21, 790–801. doi: 10.1111/jonm.12127 23865931

[ref20] Wang Z. Liu Z. Lu J. (2026). Perceived organizational support and change fatigue in nurses’ innovative behavior: a cross-sectional study in Sichuan, China. BMC Nurs. 25:278. doi: 10.1186/s12912-026-04419-z, 41715127 PMC13020311

[ref21] Wu Y. Xiao H. Wang C. Wang X. Liu H. Li W. . (2025). Chinese adaptation and reliability and validity testing of the nurses’ social capital scale. Nurs. Res. 39, 1687–1692. doi: 10.12102/j.issn.1009-6493.2025.10.015

[ref22] Xu J. Kunaviktikul W. Akkadechanunt T. Nantsupawat A. Stark A. T. (2020). A contemporary understanding of nurses’ workplace social capital: a response to the rapid changes in the nursing workforce. J. Nurs. Manag. 28, 247–258. doi: 10.1111/jonm.12914 PMC7328727 31793081

[ref23] Yang M. Yang M. Zeng D. Guo M. Wu X. Wang Z. . (2023). Analysis of the roles of job satisfaction and proactive behavior in the relationship between nurses’ job insecurity and work engagement. Chin. J. Mod. Nurs. 29, 3296–3301. doi: 10.3760/cma.j.cn115682-20221204-05829

[ref24] Yang Z. Zhang M. Guo Y. Xie F. (2025). Chinese adaptation and reliability and validity testing of the resilience scale for nurses. Tianjin Nurs. 33, 643–646. doi: 10.3969/j.issn.1006-9143.2025.06.003

[ref25] Zeng Y. Li S. Zhan Z. Chen H. Ju X. (2026). The mediating role of emotional regulation strategies in the relationship between perceived stress and change fatigue among emergency department nurses. J. Nurs. Educ. 41, 398–404. doi: 10.16821/j.cnki.hsjx.2026.04.010

[ref26] Zhang J. (2023). Analysis of the mediating effect of coworker support on the relationship between nurses’ work engagement and proactive professional behavior. J. Nurs. Contin. Educ. 38, 1191–1195. doi: 10.16821/j.cnki.hsjx.2023.13.009

[ref27] Zhang Y. He J. (2023). Analysis of the relationships among perceived social support, sense of professional mission, professional calling, and work engagement among nurses in the cardiology department of a tertiary hospital in Chengdu. Occup. Health 39, 481–486. doi: 10.13329/j.cnki.zyyjk.2023.0093

[ref28] Zhang S. Huang Q. Luo L. Wang Y. Deng B. (2025). The mediating role of workplace social capital in the relationship between clinical nurses’ perceptions of authentic leadership and organizational silence in general hospitals. Occup. Health 41, 1214–1219. doi: 10.13329/j.cnki.zyyjk.2025.0216

[ref29] Zhang X. Peng M. Wang Y. Yin S. Guo Y. Huang Z. (2024). Chinese adaptation and reliability and validity testing of the change fatigue scale. Nurs. Res. 38, 4387–4391. doi: 10.12102/j.issn.1009-6493.2024.24.009

[ref30] Zhang Q. Zhang L. Li X. Gao M. (2026). The mediating effect of change fatigue on the relationship between nurses’ perceived organizational support and work engagement. Chin. Nurs. Manag. 26, 251–255. doi: 10.3969/j.issn.1672-1756.2026.02.017

