# Supplementary material for: The chain-mediating role of workplace social capital and resilience in the relationship between change fatigue and proactive work behavior among nurses
Source: Front Psychol. 2026 Oct 8;17:1972366. doi: 10.3389/fpsyg.2026.1972366 (PMC13686869; doi:10.3389/fpsyg.2026.1972366)
Supplement: Supplementary file 1 [file Data_Sheet_1.docx]

**Questionnaire Items and Data Coding Specification**

*Supplementary Material*

*Manuscript: The Chain-Mediating Role of Workplace Social Capital and Resilience in the Relationship Between Change Fatigue and Proactive Work Behavior Among Nurses (Manuscript ID 1972366)*

*This document specifies the instruments, items, response formats, and data coding used in the submitted SPSS dataset (N = 1,018). It is provided to document that the Proactive Work Behavior Scale was administered and scored on a 7-point scale (total score 9–63), consistent with the raw data. Age, years of service, and monthly income were open-ended items in the questionnaire and were classified into groups during analysis. Hospital level was assessed in the questionnaire.*

**1. Proactive Work Behavior Scale**

**Instrument: Proactive Work Behavior Scale (Guo, 2022; 9 items, 3 dimensions).**

Response format: 7-point Likert — 1 = Strongly Disagree; 2 = Disagree; 3 = Slightly Disagree; 4 = Neutral; 5 = Slightly Agree; 6 = Agree; 7 = Strongly Agree.

Scoring: each item is scored 1–7; the total score is the sum of the 9 items, range 9–63 (midpoint 36).

| **Dimension** | **Items (variables)** | **n items** | **Scoring** |
| --- | --- | --- | --- |
| Individual proactive behavior | b1, b2, b3 | 3 | 1–7 each |
| Team member proactive behavior | b4, b5, b6 | 3 | 1–7 each |
| Organizational member proactive behavior | b7, b8, b9 | 3 | 1–7 each |
| Total score | Sum of b1–b9 | 9 | 9–63 |

Empirical check: in the submitted dataset, variables b1–b9 take integer values from 1 to 7 only; the total score ranges from 13 to 63 with a mean ± SD of 31.78 ± 9.15. This confirms the 7-point response format (a 5-point administration would produce a different value distribution and a 9–45 range).

**2. Change Fatigue Scale**

Instrument: Change Fatigue Scale (Bernerth et al., 2011; Chinese version Zhang et al., 2024; 6 items, 1 dimension).

Response format: 7-point Likert — 1 = Strongly Disagree; 2 = Disagree; 3 = Slightly Disagree; 4 = Neutral; 5 = Slightly Agree; 6 = Agree; 7 = Strongly Agree.

Items: E1–E6; total score = sum of E1–E6, range 6–42 (midpoint 21).

**3. Resilience Scale for Nurses**

Instrument: Resilience Scale for Nurses (Park et al., 2019; Chinese version Yang et al., 2025; 19 items, 4 dimensions).

Response format: 5-point Likert — 1 = Almost never; 2 = Rarely; 3 = Sometimes; 4 = Often; 5 = Almost always.

| **Dimension** | **Items (variables)** | **n items** | **Scoring** |
| --- | --- | --- | --- |
| Personality | a1–a6 | 6 | 1–5 each |
| Situational | a7–a9 | 3 | 1–5 each |
| Relational | a10–a13 | 4 | 1–5 each |
| Philosophical | a14–a19 | 6 | 1–5 each |
| Total score | Sum of a1–a19 | 19 | 19–95 |

**4. Workplace Social Capital Scale**

Instrument: Workplace Social Capital Scale (Sheingold & Sheingold, 2013; Chinese version Wu et al., 2025; 22 items, 4 dimensions).

Response format: 5-point Likert — 1 = Almost never; 2 = Rarely; 3 = Sometimes; 4 = Often; 5 = Almost always.

| **Dimension** | **Items (variables)** | **n items** | **Scoring** |
| --- | --- | --- | --- |
| External trust, solidarity, and empowerment | c1–c9 | 9 | 1–5 each |
| Internal solidarity and trust | c10–c14 | 5 | 1–5 each |
| Participation and affiliation | c15–c19 | 5 | 1–5 each |
| Social cohesion | c20–c22 | 3 | 1–5 each |
| Total score | Sum of c1–c22 | 22 | 22–110 |

**5. Demographic Variables and Coding**

*Age, years of service, and monthly income were open-ended items in the original questionnaire and were classified into groups during analysis. Hospital level was assessed in the questionnaire (4 categories); in the analysis, Grades II Class A and II Class B or below were combined into "Grade II or lower".*

| **Variable** | **Original format** | **Coding in dataset** | **Meaning** |
| --- | --- | --- | --- |
| Hospital level | 4 categories in questionnaire | 1, 2, 3, 4 | 1 = Grade III Class A; 2 = Grade III Class B; 3 = Grade II Class A; 4 = Grade II Class B or below |
| Gender | 2 categories | 1, 2 | 1 = Male; 2 = Female |
| Age | Open-ended (years) | Classified into 1–4 | 1 = ≤25; 2 = 25–30; 3 = 30–40; 4 = >40 |
| Years of service | Open-ended (years) | Classified into 1–4 | Four groups (see study records for cutoffs) |
| Education | 2 categories | 1, 2 | 1 = Associate degree or below; 2 = Bachelor or above |
| Professional title | 3 categories | 1–3 | 1 = Junior; 2 = Intermediate; 3 = Senior |
| Employment status | 2 categories | 1, 2 | 1 = Permanent; 2 = Non-permanent |
| Monthly income | Open-ended (CNY) | Classified into 1–3 | 1 = ≤4000; 2 = 4000–8000; 3 = >8000 |
| Nurse specialist | 2 categories | 1, 2 | 1 = Yes; 2 = No |
| Willingness to work in nursing | 2 categories | 1, 2 | 1 = No; 2 = Yes |
| Clear career plan | 2 categories | 1, 2 | 1 = Yes; 2 = No |

**6. Dataset Variable Naming**

Scale item variables: E1–E6 (change fatigue); a1–a19 (resilience); c1–c22 (workplace social capital); b1–b9 (proactive work behavior). Dimension and total scores are stored in Chinese-named columns in the SPSS dataset.

**7. Statement on the Proactive Work Behavior Scale Response Format**

**The Proactive Work Behavior Scale was administered and scored on a 7-point Likert format (1–7), with a total score range of 9–63. The raw coding and all analyses correspond to this 7-point version. The description of a 5-point format with a 9–45 range in the original submission was a reporting error only; no data or scoring was changed.**
