# Supplementary material for: The chain-mediating role of workplace social capital and resilience in the relationship between change fatigue and proactive work behavior among nurses
Source: Front Psychol. 2026 Oct 8;17:1972366. doi: 10.3389/fpsyg.2026.1972366 (PMC13686869; doi:10.3389/fpsyg.2026.1972366)
Supplement: Supplementary file 2 [file Data_Sheet_2.docx]

**护士变革疲劳、工作场所社会资本、复原力与工作主动行为调查问卷**Questionnaire on Nurses’ Change Fatigue, Workplace Social Capital, Resilience and Proactive Work Behavior

本文件为实际发放给临床护士填写的问卷电子档原貌。The following is the questionnaire as it was actually distributed to clinical nurses.

尊敬的护士同仁：

您好！本次问卷旨在了解临床护士变革疲劳、社会资本、复原力及工作主动行为现状。问卷所有答案仅用于学术研究，严格保密个人信息，数据仅做群体统计分析，不做个体评价。问卷匿名填写，大约需要 8 分钟完成，请您根据自身真实工作感受填写。感谢您的支持！

Dear nursing colleagues: This anonymous survey concerns nurses’ change fatigue, social capital, resilience, and proactive work behavior. All answers are for academic research only, personal information is kept strictly confidential, and only group-level analyses are performed. Completion takes about 8 minutes. Thank you for your support!By completing this questionnaire, you provide informed consent to participate in this study. Thank you for your participation!

**第一部分 一般资料（General Information）**

请根据您的实际情况选择或填写相应选项。（Please select or fill in the option that describes you.）

1. 医院级别（Hospital level）：□ 三级甲等（Grade III Class A）　□ 三级乙等（Grade III Class B）　□ 二级甲等（Grade II Class A）　□ 二级乙等及以下（Grade II Class B or below）

2. 性别（Gender）：□ 男（Male）　□ 女（Female）

3. 年龄（Age）：________岁（years）

【注】原始问卷为开放题，分析时按 ≤25 / 25–30 / 30–40 / >40 四组归类。

Note: open-ended in the questionnaire; classified into ≤25 / 25–30 / 30–40 / >40 during analysis.

4. 工龄（Years of service）：________年（years）

【注】原始问卷为开放题，分析时归类为 4 组（具体年限见研究记录）。

Note: open-ended in the questionnaire; classified into 4 groups during analysis (see study records for cutoffs).

5. 受教育程度（Education）：□ 大专及以下（Associate degree or below）　□ 本科及以上（Bachelor or above）

6. 职称（Professional title）：□ 初级（Junior）　□ 中级（Intermediate）　□ 高级（Senior）

7. 聘任形式（Employment status）：□ 在编（Permanent）　□ 非在编（Non-permanent）

8. 个人月均收入（Monthly income，元）：________元

【注】原始问卷为开放题，分析时按 ≤4000 / 4000–8000 / >8000 三组归类。

Note: open-ended in the questionnaire; classified into ≤4000 / 4000–8000 / >8000 during analysis.

9. 是否专科护士（Nurse specialist）：□ 是（Yes）　□ 否（No）

10. 是否愿意从事护理工作（Willingness to work in nursing）：□ 否（No）　□ 是（Yes）

11. 是否有明确的职业发展规划（Clear career plan）：□ 有（Yes）　□ 无（No）

**第二部分 变革疲劳量表（Change Fatigue Scale）**

请根据您的实际感受，选择最符合的一项。1 = 非常不同意，2 = 不同意，3 = 有点不同意，4 = 中立，5 = 有点同意，6 = 同意，7 = 非常同意。

Response format: 7-point Likert (1 = Strongly Disagree; 2 = Disagree; 3 = Slightly Disagree; 4 = Neutral; 5 = Slightly Agree; 6 = Agree; 7 = Strongly Agree).

| **条目** | **非常不同意 （1）** | **不同意 （2）** | **有点不同意 （3）** | **中立 （4）** | **有点同意 （5）** | **同意（6）** | **非常同意 （7）** |
| --- | --- | --- | --- | --- | --- | --- | --- |
| 1本机构实施了太多变革举措 | □ | □ | □ | □ | □ | □ | □ |
| 2我已经厌倦本机构的各类变革 | □ | □ | □ | □ | □ | □ | □ |
| 3本机构发生的变革幅度很大 | □ | □ | □ | □ | □ | □ | □ |
| 4机构要求我们做出的改变太多 | □ | □ | □ | □ | □ | □ | □ |
| 5感觉我们总是被要求不断做出改变 | □ | □ | □ | □ | □ | □ | □ |
| 6在开展新变革前，我希望能有一段稳定时期 | □ | □ | □ | □ | □ | □ | □ |

**第三部分 护士心理韧性量表（Resilience Scale for Nurses）**

请根据您的实际情况，选择最符合的一项。1 = 几乎没有，2 = 很少，3 = 有时，4 = 经常，5 = 几乎总是。

Response format: 5-point Likert (1 = Almost never; 2 = Rarely; 3 = Sometimes; 4 = Often; 5 = Almost always).

| **条目** | **几乎没有（1）** | **很少 （2）** | **有时（3）** | **经常（4）** | **几乎总是（5）** |
| --- | --- | --- | --- | --- | --- |
| 1. 面对压力事件时，我性格上能够保持稳定 | □ | □ | □ | □ | □ |
| 2. 我能够接纳自身的缺点，不轻易自我否定 | □ | □ | □ | □ | □ |
| 3. 遇到挫折时，我拥有较强的内在意志力 | □ | □ | □ | □ | □ |
| 4. 我倾向于用积极的视角看待困难 | □ | □ | □ | □ | □ |
| 5. 遇到困境时，我不容易产生长期的自我怀疑 | □ | □ | □ | □ | □ |
| 6. 我能够调控自身负面情绪，不被情绪裹挟 | □ | □ | □ | □ | □ |
| 7. 面对突发工作难题，我可以快速适应新情境 | □ | □ | □ | □ | □ |
| 8. 当工作环境发生变化，我能快速调整应对方式 | □ | □ | □ | □ | □ |
| 9. 遭遇工作挫折后，我能够较快从困境中恢复 | □ | □ | □ | □ | □ |
| 10. 遇到压力时，我能从同事那里获得情感支持 | □ | □ | □ | □ | □ |
| 11. 家人能够理解我临床工作的压力并给予支持 | □ | □ | □ | □ | □ |
| 12. 我愿意向身边信任的人倾诉工作压力 | □ | □ | □ | □ | □ |
| 13. 良好的人际关系能够帮助我渡过困难时期 | □ | □ | □ | □ | □ |
| 14. 我认为困难和挫折是工作中正常的一部分 | □ | □ | □ | □ | □ |
| 15. 困境能够促使我成长，积累工作经验 | □ | □ | □ | □ | □ |
| 16. 我相信大部分困境最终都会得到改善 | □ | □ | □ | □ | □ |
| 17. 即便遇到失败，我依然保有对未来的希望 | □ | □ | □ | □ | □ |
| 18. 我认为人有能力克服大部分困难 | □ | □ | □ | □ | □ |
| 19. 困难经历会让我更加珍惜平稳的工作状态 | □ | □ | □ | □ | □ |

**第四部分 工作场所社会资本量表（Workplace Social Capital Scale）**

请根据您的实际情况，选择最符合的一项。1 = 几乎没有，2 = 很少，3 = 有时，4 = 经常，5 = 几乎总是。

Response format: 5-point Likert (1 = Almost never; 2 = Rarely; 3 = Sometimes; 4 = Often; 5 = Almost always).

| **条目** | **几乎没有（1）** | **很少 （2）** | **有时 （3）** | **经常（4）** | **几乎总是（5）** |
| --- | --- | --- | --- | --- | --- |
| 1科室护士群体能够获得医院管理层面的信任 | □ | □ | □ | □ | □ |
| 2医院管理方愿意听取护士群体提出的意见 | □ | □ | □ | □ | □ |
| 3医院给予护士参与科室管理的机会 | □ | □ | □ | □ | □ |
| 4医院重视护士团队的共同目标与集体利益 | □ | □ | □ | □ | □ |
| 5护士群体在医院内部拥有一定话语权 | □ | □ | □ | □ | □ |
| 6医院支持护士团队协同解决临床工作难题 | □ | □ | □ | □ | □ |
| 7护士群体能够感受到医院赋予的工作自主权 | □ | □ | □ | □ | □ |
| 8医院管理与护士群体之间保持良好沟通 | □ | □ | □ | □ | □ |
| 9医院能够尊重护士群体的集体诉求 | □ | □ | □ | □ | □ |
| 10科室内部护士之间彼此信任 | □ | □ | □ | □ | □ |
| 11科室同事之间能够真诚沟通、坦诚交流 | □ | □ | □ | □ | □ |
| 12科室护士之间互相尊重 | □ | □ | □ | □ | □ |
| 13遇到困难时，科室同事愿意互相帮助 | □ | □ | □ | □ | □ |
| 14科室内部护士能够团结协作完成工作 | □ | □ | □ | □ | □ |
| 15我有归属感，认同自己属于本科室护理团队 | □ | □ | □ | □ | □ |
| 16我愿意参与科室组织的团队讨论和事务决策 | □ | □ | □ | □ | □ |
| 17科室重要事项会征求我们一线护士的意见 | □ | □ | □ | □ | □ |
| 18我关心科室护理团队的整体发展 | □ | □ | □ | □ | □ |
| 19我愿意为科室团队目标贡献自己力量 | □ | □ | □ | □ | □ |
| 20科室护士之间关系融洽，团队凝聚力强 | □ | □ | □ | □ | □ |
| 21科室成员拥有共同的团队价值追求 | □ | □ | □ | □ | □ |
| 22科室护士能够互相扶持，共渡工作难关 | □ | □ | □ | □ | □ |

**第五部分 前瞻性工作行为量表（Proactive Work Behavior Scale）★**

请根据您的实际工作行为，选择最符合的一项。1 = 非常不同意，2 = 不同意，3 = 有点不同意，4 = 中立，5 = 有点同意，6 = 同意，7 = 非常同意。

Response format: 7-point Likert (1 = Strongly Disagree; 2 = Disagree; 3 = Slightly Disagree; 4 = Neutral; 5 = Slightly Agree; 6 = Agree; 7 = Strongly Agree).

本量表共 9 个条目，采用 7 点计分，总分范围 9–63。

| **条目** | **非常不同意 （1）** | **不同意 （2）** | **有点不同意 （3）** | **中立 （4）** | **有点同意 （5）** | **同意（6）** | **非常同意 （7）** |
| --- | --- | --- | --- | --- | --- | --- | --- |
| 1我主动思考改进个人本职护理工作的方法 | □ | □ | □ | □ | □ | □ | □ |
| 2我主动寻找办法提升自身护理工作效率 | □ | □ | □ | □ | □ | □ | □ |
| 3我主动调整工作方式，解决个人工作中的问题 | □ | □ | □ | □ | □ | □ | □ |
| 4我主动提出改善科室护理团队工作效率的建议 | □ | □ | □ | □ | □ | □ | □ |
| 5我主动协助科室优化护理工作流程 | □ | □ | □ | □ | □ | □ | □ |
| 6我主动推动科室团队内部的工作改进 | □ | □ | □ | □ | □ | □ | □ |
| 7我主动提出有利于医院护理发展的改进想法 | □ | □ | □ | □ | □ | □ | □ |
| 8我主动推动医院层面护理相关的优化变革 | □ | □ | □ | □ | □ | □ | □ |
| 9我主动识别医院护理管理、流程中存在的问题 | □ | □ | □ | □ | □ | □ | □ |

**您的其他建议（Other suggestions, optional）**

__________________________________________________________________________________

__________________________________________________________________________________

__________________________________________________________________________________
