# Supplementary material for: The chain-mediating role of workplace social capital and resilience in the relationship between change fatigue and proactive work behavior among nurses
Source: Front Psychol. 2026 Oct 8;17:1972366. doi: 10.3389/fpsyg.2026.1972366 (PMC13686869; doi:10.3389/fpsyg.2026.1972366)
Supplement: Supplementary file 3 [file Data_Sheet_3.docx]

**Supplementary File 1 — Administered Questionnaire (English translation of the Chinese questionnaire distributed to participants; response formats and scoring identical to the original).**

**Administered Questionnaire**

*(English translation of the Chinese questionnaire distributed to participants)*

*The questionnaire was administered in Chinese; this is an English translation provided for review purposes. The response formats and scoring are identical to the original.*

**Dear nursing colleagues:**

*This anonymous survey concerns nurses’ change fatigue, social capital, resilience, and proactive work behavior. All answers are for academic research only, personal information is kept strictly confidential, and only group-level analyses are performed. Completion takes about 8 minutes. Thank you for your support!*

**Part 1. General Information**

Please select or fill in the option that describes you.

1. Hospital level: □ Grade III Class A □ Grade III Class B □ Grade II Class A □ Grade II Class B or below

*Note: assessed in the questionnaire (4 categories); in the analysis, Grades II Class A and II Class B or below were combined into "Grade II or lower".*

2. Gender: □ Male □ Female

3. Age: ________ years

*Note: open-ended in the questionnaire; classified into ≤25 / 25–30 / 30–40 / >40 during analysis.*

4. Years of service: ________ years

*Note: open-ended in the questionnaire; classified into 4 groups during analysis.*

5. Education: □ Associate degree or below □ Bachelor or above

6. Professional title: □ Junior □ Intermediate □ Senior

7. Employment status: □ Permanent □ Non-permanent

8. Monthly income (CNY): ________

*Note: open-ended in the questionnaire; classified into ≤4000 / 4000–8000 / >8000 during analysis.*

9. Nurse specialist: □ Yes □ No

10. Willingness to work in nursing: □ No □ Yes

11. Clear career plan: □ Yes □ No

**Part 2. Change Fatigue Scale**

Please select the option that best describes your feelings. 1 = Strongly Disagree; 2 = Disagree; 3 = Slightly Disagree; 4 = Neutral; 5 = Slightly Agree; 6 = Agree; 7 = Strongly Agree.

1. This institution has implemented too many change initiatives.

2. I am already tired of the various changes in this institution.

3. The magnitude of changes occurring in this institution is large.

4. The institution requires us to make too many changes.

5. I feel that we are always being asked to keep making changes.

6. I hope there can be a period of stability before new changes are launched.

**Part 3. Resilience Scale for Nurses**

Please select the option that best describes you. 1 = Almost never; 2 = Rarely; 3 = Sometimes; 4 = Often; 5 = Almost always.

1. When facing stressful events, I can remain stable in my disposition. [Personality]

2. I can accept my own shortcomings without readily denying myself. [Personality]

3. When encountering setbacks, I have strong inner willpower. [Personality]

4. I tend to view difficulties from a positive perspective. [Personality]

5. When facing adversity, I am not prone to prolonged self-doubt. [Personality]

6. I can regulate my negative emotions without being overwhelmed by them. [Personality]

7. Facing unexpected work problems, I can quickly adapt to new situations. [Situational]

8. When the work environment changes, I can quickly adjust my coping strategies. [Situational]

9. After work setbacks, I can recover from difficulties relatively quickly. [Situational]

10. When under pressure, I can obtain emotional support from colleagues. [Relational]

11. My family understands the pressure of my clinical work and gives me support. [Relational]

12. I am willing to confide work-related stress to people I trust. [Relational]

13. Good interpersonal relationships can help me get through difficult times. [Relational]

14. I believe difficulties and setbacks are a normal part of work. [Philosophical]

15. Adversity can promote my growth and help me accumulate work experience. [Philosophical]

16. I believe most difficulties will eventually improve. [Philosophical]

17. Even when I encounter failure, I still retain hope for the future. [Philosophical]

18. I believe people are capable of overcoming most difficulties. [Philosophical]

19. Difficult experiences make me value a stable work situation more. [Philosophical]

**Part 4. Workplace Social Capital Scale**

Please select the option that best describes you. 1 = Almost never; 2 = Rarely; 3 = Sometimes; 4 = Often; 5 = Almost always.

1. The nursing group in the department can gain the trust of hospital management. [External]

2. Hospital management is willing to listen to the opinions raised by nurses. [External]

3. The hospital gives nurses opportunities to participate in department management. [External]

4. The hospital values the common goals and collective interests of the nursing team. [External]

5. The nursing group has a certain voice within the hospital. [External]

6. The hospital supports the nursing team in collaboratively solving clinical work problems. [External]

7. Nurses can feel the work autonomy granted by the hospital. [External]

8. Hospital management maintains good communication with the nursing group. [External]

9. The hospital respects the collective demands of the nursing group. [External]

10. Nurses within the department trust each other. [Internal]

11. Colleagues in the department can communicate sincerely and openly. [Internal]

12. Nurses in the department respect each other. [Internal]

13. When facing difficulties, colleagues in the department are willing to help each other. [Internal]

14. Nurses in the department can work together in unity to complete tasks. [Internal]

15. I have a sense of belonging and identify with the nursing team of my department. [Participation]

16. I am willing to participate in team discussions and decision-making organized by the department. [Participation]

17. The department seeks the opinions of frontline nurses on important matters. [Participation]

18. I care about the overall development of the department’s nursing team. [Participation]

19. I am willing to contribute to the department team’s goals. [Participation]

20. The relationship among nurses in the department is harmonious and the team is highly cohesive. [Cohesion]

21. Department members share common team values. [Cohesion]

22. Nurses in the department can support each other through work difficulties. [Cohesion]

**Part 5. Proactive Work Behavior Scale**

**Please select the option that best describes your actual work behavior. 1 = Strongly Disagree; 2 = Disagree; 3 = Slightly Disagree; 4 = Neutral; 5 = Slightly Agree; 6 = Agree; 7 = Strongly Agree.**

**This scale has 9 items, uses a 7-point response format, and the total score ranges from 9 to 63.**

1. I proactively think of ways to improve my own nursing work. [Individual]

2. I proactively seek ways to improve my nursing work efficiency. [Individual]

3. I proactively adjust my working methods to solve problems in my own work. [Individual]

4. I proactively put forward suggestions to improve the work efficiency of the department’s nursing team. [Team member]

5. I proactively assist the department in optimizing nursing work processes. [Team member]

6. I proactively promote work improvements within the department team. [Team member]

7. I proactively put forward improvement ideas that benefit the hospital’s nursing development. [Organizational]

8. I proactively promote nursing-related optimization and changes at the hospital level. [Organizational]

9. I proactively identify problems in hospital nursing management and processes. [Organizational]

**Other suggestions (optional)**

___________________________________________________________________________________

___________________________________________________________________________________

___________________________________________________________________________________
