# Supplementary material for: The chain-mediating role of workplace social capital and resilience in the relationship between change fatigue and proactive work behavior among nurses
Source: Front Psychol. 2026 Oct 8;17:1972366. doi: 10.3389/fpsyg.2026.1972366 (PMC13686869; doi:10.3389/fpsyg.2026.1972366)
Supplement: Supplementary file 4 [file Table_1.docx]

| Construct | Indicator (subscale / item) | Standardized loading |
| --- | --- | --- |
| Change Fatigue | E1 | 0.896 |
| Change Fatigue | E2 | 0.929 |
| Change Fatigue | E3 | 0.931 |
| Change Fatigue | E4 | 0.963 |
| Change Fatigue | E5 | 0.973 |
| Change Fatigue | E6 | 0.943 |
| Workplace Social Capital | External trust/solidarity/empowerment | 0.903 |
| Workplace Social Capital | Internal solidarity/trust | 0.841 |
| Workplace Social Capital | Participation/affiliation | 0.990 |
| Workplace Social Capital | Social cohesion | 0.848 |
| Resilience | Personality | 0.696 |
| Resilience | Situational | 0.738 |
| Resilience | Relational | 0.764 |
| Resilience | Philosophical | 0.604 |
| Proactive Work Behavior | Individual | 0.876 |
| Proactive Work Behavior | Team member | 0.938 |
| Proactive Work Behavior | Organizational | 0.962 |
